# Supplementary material for: Ecological patterns in the Porto-Novo Lagoon (Benin, West Africa): A review with implications for SDG 6.3.2 and EU-WFD readiness toward ecological status classification
Source: PLoS One. 2026 Apr 28;21(4):e0348113. doi: 10.1371/journal.pone.0348113 (PMC13123969; doi:10.1371/journal.pone.0348113)
Supplement: S1 File — This file contains additional methodological notes, framework requirements, calculation procedures, completeness-score explanations, and acronym definitions. (DOCX) [file pone.0348113.s001.docx]

# **Supplementary material**

**Ecological classification of the Porto-Novo Lagoon (Benin): feasibility SDG 6.3.2 and adapting the EU Water Framework Directive in a West African context**.

### **S1. Minimum formal requirements of the two reference frameworks**

**This table distils the formal requirements of the two reference frameworks used in the manuscript—SDG 6.3.2 (UNEP GEMS/Water) and the EU Water Framework Directive (WFD)—covering mandatory variables, minimum sampling designs and the logic of the final classification. It complements the overview presented in the Introduction (“International frameworks…”) and clarifies how Section 3.4 interprets dataset completeness against each framework’s minimum thresholds.**

| **Framework** | **Set of obligatory variables** | **Minimum frequency and duration** | **Classification method** | **Key references** |
| --- | --- | --- | --- | --- |
| **UNEP GEMS/Water – SDG 6.3.2** | 5 physico-chemical parameters:  • pH  • Dissolved oxygen  (DO)  • Conductivity (EC)  • Biochemical oxygen  demand (BOD₅)  • Ammoniacal nitrogen or nitrite (NH₃-N / NO₂- N) | • ≥ 10 measurements per parameter over the reporting period (5 rolling years)  • Minimum: sampling every six months (but monthly recommended)  • At least one representative station per water body (≥ 3 stations recommended) | Each value is classified into one of four classes I → IV (GEMS thresholds).  The water body is declared to have *Good Ambient Water Quality* when ≥ 80% of measurements fall into classes I or II (final binary result: good / not good). | UN Environment Programme (2020); UN Water (2018) |
| **EU Water** **Framework** **Directive** **(WFD** **2000 / 60 / EC)** | • **Biological Quality** **Elements (BQE)**:  – Phytoplankton (biomass, composition, blooms)  – Macrophytes /macroalgae  – Benthic macroinvertebrates  – Fish (indicative for transitional waters)  • **Supporting physico-chemistry**: nutrients (N, P), oxygen, transparency, temperature, salinity  • **Hydromorphology: hydrological regime (discharge, tide), continuity, morphology of the riverbed and banks** | • «Trend» monitoring: once per month for nutrients and DO, once per quarter for metals; biological monitoring at least once per year (phytoplankton: three times per year).  • Multi-year series (management cycle: 6 years).  • Reference data required for each BQE (minimally disturbed or best available sites). | Calculation of an Ecological Quality Ratio (EQR) for each BQE (0-1).  Very good / good / average / poor / bad status is determined by comparison with the reference; the "one-out, all-out" principle applies the most degraded class to the whole. | Directive 2000/60/EC; European Commission Guidance No 13 (2005); CIS Doc. No 5 (2003). |

**Key points about the framework:**

• **SDG 6.3.2 focuses exclusively on basic physical chemistry; it does not require biota or hydromorphology and delivers a good/not good verdict once the five parameters have been monitored over five years.**

**• WFD is much more demanding: the assessment can only be carried out if all BQEs, supporting physico-chemistry and hydromorphological elements have at least one multi-year data set and a validated reference condition for the water body in question.**

**• In the West African context, the availability of a continuous series for the five SDG parameters is already a challenge; the absence of "pristine" sites further complicates the establishment of the references necessary for WFD classification, particularly for BQEs and hydromorphology.**

### **S2 Official procedure for calculating the "WFD" index**

This note summarises the official steps for computing a WFD-compliant ecological status: defining BQE metrics, establishing type-specific reference conditions, scaling to the 0–1 Ecological Quality Ratio (EQR) and applying intercalibrated class boundaries under the “one-out, all-out” rule. It provides the methodological background for the feasibility discussion in Section 4.2 (“WFD: feasible in the medium/long term after structural prerequisites?”).

A "complete WFD" index (as defined by the Water Framework Directive – WFD) meets four criteria simultaneously:

1. Clearly defined metric variable(s) for a biological quality element (BQE).

2. Reference conditions established for the type of water body (river, lake, transitional water, coastal water).

3. Continuous Ecological Quality Ratio (EQR) scale normalised 0–1 and intercalibrated limits between Member States for the five status classes (very good–poor).

4. Official intercalibration procedure published in a "CIS Guidance / Intercalibration Decision" document or recognised journal.

The table below lists the main indices currently considered comprehensive for each of the major categories of EQI, with a focus on transitional water bodies (lagoons, estuaries) closest to the Porto-Novo context.

| **BQE (WFD)** | **Water category** | **Complete WFD index** | **Intercalibration references*** |
| --- | --- | --- | --- |
| Phytoplankton | Transitional / coastal waters | - ICM-Phytoplankton (biovolume, % cyanobacteria, bloom frequency); - EQR- Chl-Lagoon (average chlorophyll-a in dry and wet seasons) | European Commission 2013a; Devlin et al. 2014 |
| Macroalgae /  Macrophytes | Transitional waters | - EEI (Ecological Evaluation Index) for benthic macroalgae - Trophic Index (TI) for lagoon seagrass beds | Orfanidis *et al.* 2011; EC 2015 |
| Benthic  macroinvertebrates | Estuaries and  lagoons | - M-AMBI (Multivariate AZTI Marine Biotic Index); - EQR-AMBI (version harmonisée) | Borja et Tunberg 2011; EC 2013b |
| Fish | Transitional waters | - EFSI (Estuarine Fish Status Index) - Transitional Fish Classification Index – TFCI | Borja *et al.* 2012; Franco *et al.* 2020 |
| Diatoms | River and Estuaries | - DBI (Diatom Biological Index); - TDI (Trophic Diatom Index) | Kelly *et al.* 2014 |
| Hydromorphology (supporting) | All types | - HYMO-EQR (Regime Flow Index + Morphology Index) – rivières - HET (Hydromorphological Evaluation Tool) – eaux de transition | EC Guidance No 37 (2017) |

### **S3 Official procedure for calculating the binary indicator "Good Ambient Water Quality" (SDG 6.3.2)**

This step-by-step summary details the SDG 6.3.2 computation from station selection and five-year windowing to per-parameter pass/fail classification and aggregation to a binary water-body outcome. It supports Section 4.2 (“SDG 6.3.2: feasible in the short term under conditions?”), where windowing and hybrid data strategies are discussed for the Porto-Novo context.

| **Step** | **Operational description** | **Key points/remarks** |
| --- | --- | --- |
| 1. Delimit water bodies | Each lake, lagoon, estuary or river section is defined as a "Water Body" (WB) – the minimum reporting unit. | For a country, the final result is the percentage of WBs in 'good' status. |
| 2. Select monitoring station(s) | At least one representative station per WB; several stations if there are marked gradients. | The station must reflect the "most representative" quality, not necessarily the most degraded point. |
| 3. Collect data | • Period: five rolling years.  • Minimum frequency: ≥ 2 samples/year (i.e. ≥ 10 results/parameter over 5 years).  • Mandatory parameters: 1. pH, 2. Dissolved oxygen (DO), 3. Conductivity (EC), 4. Biochemical oxygen demand (BOD₅), 5. Ammoniacal nitrogen or nitrite (NH₃-N / NO₂-N). | In situ analysis or accredited laboratory, ISO/SM methods recommended. |
| 4. Set reference thresholds | Two options:  • National thresholds (where available);  • Otherwise, UNEP GEMS/Water Class I–II thresholds (GEMS Tables 2017). | Class III–IV thresholds represent a "fair to poor" status. |
| 5. Classify  each value | Compare each result to the threshold:  • If compliant → "acceptable" (class I or II).  • Otherwise → "unacceptable" (class III or IV). | For pH, the range 6–9 is considered "acceptable" by default in GEMS. |
| 6. Calculate the proportion of "acceptable" | For each parameter (independently):  Pi= (Number of acceptable measurements Total/ measurements) ×100 |  |
| 7. Parameter status | Parameter i is considered « good » if Pi ≥ 80 % |  |
| 8. Status of the water body | The WB is classified as "Good Ambient Water Quality" if all five parameters are "good". Otherwise → "Not Good". | One-out, all-out rule applied to the 5 parameters. |
| 9. National aggregation | Indicateur SDG 6.3.2(%) = [NWB «good»] / [NWB «evaluated»] x 100.  NWB«good» is the number of water bodies (WB) classified as ‘good ambient water quality  NWB«evaluated» the total number of water bodies assessed over the reference period. | The result is expressed as a percentage of water bodies in "good" condition. |
| 10.Management of gaps | If a parameter is missing:  • Option 1: classify WB as "Not Good" (cautious approach);  • Option 2: report WB as "Not assessed". | The UN encourages filling in the missing data first. |

Simplified example for a lagoon monitored from 2018 to 2022:

- pH: 12/12 "acceptable" values → 100% → Good.
- DO: 20/24 values → 83% → Good.
- EC: 19/24 values → 79% → Not Good (because < 80%).

Even though the other three parameters are "good", the lagoon will be classified as Not Good under the *one-out, all-out* rule.

### **S4 Explanation of the data in Figure 4.1: Degree of completeness (%) of the Porto-Novo lagoon datasets with regard to SDG 6.3.2 and WFD requirements**

This note explains how the completeness percentages reported in Figure 4.1 in the manuscript were derived for SDG 6.3.2 and WFD: scoring rules per parameter/family, treatment of temporal continuity (≥ 5 consecutive years), and handling of spatial coverage. It documents the assumptions behind Section 3.4 and the summary values discussed in the Results/Discussion.

The following is a summary (Figure 6) of the level of data completeness for each evaluation framework: National thresholds (where applicable)

- 60% of the basic requirements of SDG 6.3.2 are currently covered by the literature (pH, DO, EC), but gaps in BOD₅ and NH₃/NO₂ still limit the calculation of the indicator.
- Supporting physicochemical data in accordance with the WFD reaches 55%: discontinuous series, no tropical reference conditions.
- Biological elements (BQE) do not exceed 40%: only phytoplankton and ichthyofauna have usable series, without a standardised WFD index.
- Hydromorphology remains the weakest link (20%), due to the lack of a formalised index for the hydraulic regime, continuity and integrity of the banks.

This visualisation confirms the urgent need to extend monitoring to the missing parameters (BOD₅, NH₃/NO₂) and to develop tropical indices for benthic communities and hydromorphology before any attempt at official classification.

### **S5 Definitions of acronyms mentioned in the "Implementing authority" column (Table 3.1)**

This glossary expands the acronyms cited in the “Implementing authority” column of **Table 3.1** (legal and institutional frameworks across the four lagoons). It ensures unambiguous attribution of roles when comparing governance settings referenced in Sections 3.4 and 4.3.

| **Acronym** | **Full name** | **Country** | **Main role** |
| --- | --- | --- | --- |
| **DSIDH** | *Directorate of Hydraulic Surveillance, Inspection and Documentation* | Benin | Technical department responsible for monitoring and controlling resources within the DG-Water. |
| **DG-Water** | *Directorate-General for Water*  (Ministry of Water and Mines) | Benin | National Authority for Planning and Regulation of the Water Sector. |
| **NESREA** | *National Environmental Standards and Regulations Enforcement Agency* | Nigeria | Federal agency responsible for enforcing environmental standards (water, air, soil). |
| **EPA- Ghana** | *Environmental Protection Agency, Ghana* | Ghana | State institution responsible for regulating and monitoring environmental quality, including surface water. |
| **WRC** | *Water Resources Commission* | Ghana | Independent body that allocates water rights, coordinates and monitors the sustainable use of water resources. |
| **ONAD** | *National Office for Sanitation and*  *Drainage* | Côte d'Ivoire | Public body responsible for wastewater management, storm drainage and monitoring of urban water bodies (Ébrié lagoon). |
| **ANDE** | *National Environment Agency* | Côte d'Ivoire | Competent authority for environmental assessment and control, including water quality  monitoring. |

These definitions supplement Table 4.1 and specify the institutions responsible for implementing legislation relating to lagoon water quality.

### **References**

Borja, Á. et Tunberg, B. (2011) ‘Assessing benthic ecological status in disturbed estuarine and coastal waters’, *Marine Pollution Bulletin*, 62, 2185-2197.

Borja, Á., Franco, J. et Elliott, M. (2012) ‘A fish-based index of estuarine ecological quality’, *Ecological Indicators*, 23, 524-537.

Devlin, M.J., Best, M. et Co-authors (2014) ‘Phytoplankton metrics for the European transition-water quality assessment’, *Ecological Indicators*, 49, 1-13.

European Commission (2003) Common Implementation Strategy Guidance Document No 5: Transitional and Coastal Waters – Typology, Reference Conditions and Classification Systems. Luxembourg: Publications Office of the European Union.

European Commission (2005) Guidance Document No 13: Overall Approach to the Classification of Ecological Status and Ecological Potential. Luxembourg: Publications Office of the European Union.

European Commission (2013a) *Decision 2013/480/EU establishing the values of the Member States’ ecological classification… for phytoplankton in coastal and transitional waters*.

European Commission (2013b) *Decision 2013/481/EU… for benthic invertebrates in coastal and transitional waters*.

European Commission (2015) *CIS Guidance No 30: Macrophytes and Angiosperms in Transitional Waters*.

European Commission (2017) *CIS Guidance No 37: Hydromorphological assessment of coastal and transitional waters*.

European Parliament and Council (2000) Directive 2000/60/EC establishing a framework for Community action in the field of water policy. OJ L327, 22 December 2000.

Franco, J. et al. (2020) ‘Improving the Transitional Fish Classification Index (TFCI)’, *Ecological Indicators*, 114, 106265.

Kelly, M.G., Juggins, S. et Co-authors (2014) ‘Taking account of uncertainty in WFD classification of river diatom status’, *Ecological Indicators*, 44, 507-517.

Orfanidis, S., Panayotidis, P. et Stamatis, N. (2011) ‘An insight to the Ecological Evaluation Index (EEI)’, *Ecological Indicators*, 11, 1516-1522.

UN Environment Programme (2020) Water Quality Monitoring for SDG 6.3.2 – Step-by-Step Methodology (Version 2). Nairobi: UNEP/GEMS Water.

UN-Water (2018) Progress on Ambient Water Quality – SDG 6 Indicator 6.3.2. Geneva: UN-Water.
