# Supplementary material for: Ecological patterns in the Porto-Novo Lagoon (Benin, West Africa): A review with implications for SDG 6.3.2 and EU-WFD readiness toward ecological status classification
Source: PLoS One. 2026 Apr 28;21(4):e0348113. doi: 10.1371/journal.pone.0348113 (PMC13123969; doi:10.1371/journal.pone.0348113)
Supplement: S2 Checklist — (DOCX) [file pone.0348113.s002.docx]

**Preferred Reporting Items for Systematic reviews and Meta-Analyses extension for Scoping Reviews (PRISMA-ScR) Checklist**

| **SECTION** | **ITEM** | **PRISMA-ScR CHECKLIST ITEM** | **REPORTED ON PAGE #** |
| --- | --- | --- | --- |
| TITLE | 1 | Identify the report as a scoping review. | 1; 6 |
| ABSTRACT | 2 | Provide a structured summary (background, objectives, eligibility criteria, sources of evidence, charting methods, results, conclusions). | 1 |
| INTRODUCTION | 3 | Describe the rationale for the review in the context of what is already known. | 3 |
|  | 4 | Provide an explicit statement of the questions and objectives being addressed with reference to key elements (e.g., population/participants, concept, context). | 3–4 |
| METHODS | 5 | Indicate whether a review protocol exists (e.g., registration), and where it can be accessed. | N/A (not registered) |
|  | 6 | Specify eligibility criteria (e.g., years, language, types of sources of evidence). | 7 |
|  | 7 | Describe all information sources (e.g., databases, registers) with dates of coverage. | 6 |
|  | 8 | Present the full electronic search strategy for at least one database, including any limits used. | 6 |
|  | 9 | State the process for selecting sources of evidence (screening, eligibility, included). | 7 |
|  | 10 | Describe the data charting process (e.g., calibration of forms; whether charting was done independently/duplicated). | 7 |
|  | 11 | List and define all variables for which data were sought (data items). | 7 |
|  | 12 | If done, describe methods for critical appraisal of included sources of evidence. | 7 |
|  | 13 | Describe methods of handling and summarizing the data (synthesis). | 7 |
| RESULTS | 14 | Provide numbers of sources screened, assessed for eligibility, and included, with reasons for exclusions (ideally with a flow diagram). | Not reported in the manuscript |
|  | 15 | For each included source of evidence, present characteristics of the sources of evidence. | 14–20 |
|  | 16 | If done, present data on critical appraisal of included sources of evidence. | Not performed |
|  | 17 | Present the results of individual sources of evidence as relevant to the review question(s). | 8–17 |
|  | 18 | Summarize and/or present the charting results in relation to the review question(s) and objectives. | 14; 18–21 |
| DISCUSSION | 19 | Summarize the main results, including an overview of concepts, themes, and types of evidence available. | 18–21 |
|  | 20 | Discuss limitations of the scoping review process. | 21–22 |
|  | 21 | Provide a general interpretation of the results with respect to the review questions and objectives, as well as potential implications and/or next steps. | 24–25 |
| FUNDING | 22 | Describe sources of funding for the included sources of evidence and for the scoping review; describe the role of the funders. | N/A (no specific funding) |
